# Modification of EBV-Associated Pathologies and Immune Control by Coinfections

**DOI:** 10.3389/fonc.2021.756480

**Published:** 2021-10-28

**Authors:** Christian Münz

**Affiliations:** Viral Immunobiology, Institute of Experimental Immunology, University of Zürich, Zürich, Switzerland

**Keywords:** Burkitt’s lymphoma, primary effusion lymphoma, cytotoxic lymphocytes, humanized mice, diffuse large B cell lymphoma, T cells, NK cells

## Abstract

The oncogenic Epstein–Barr virus (EBV) persistently infects more than 95% of the human adult population. Even so it can readily transform human B cells after infection *in vitro*, it only rarely causes tumors in patients. A substantial proportion of the 1% of all human cancers that are associated with EBV occurs during coinfections, including those with the malaria parasite *Plasmodium falciparum*, the human immunodeficiency virus (HIV), and the also oncogenic and closely EBV-related Kaposi sarcoma-associated herpesvirus (KSHV). In this review, I will discuss how these infections interact with EBV, modify its immune control, and shape its tumorigenesis. The underlying mechanisms reveal new aspects of EBV-associated pathologies and point toward treatment possibilities for their prevention by the human immune system.

## Introduction on EBV and Its Immune Control

The ubiquitous human γ-herpesvirus EBV persistently infects more than 95% of the human adult population (1, 2). While it is primarily acquired in early childhood, delayed primary infection during adolescence or young adulthood in one-third of the European and North American population can result in infectious mononucleosis (IM), an immunopathological CD8^+^ T cell lymphocytosis (3, 4). In some of these individuals, chronic active EBV (CAEBV) can develop which allows the virus to infect in addition to its main host cell, the human B cell, and other lymphocytes, including T and NK cells (5–7). From this EBV infection of T and NK cells, also virus-associated NK/T cell lymphomas are thought to develop (8). Most types of EBV-associated lymphomas, however, mirror the viral infection programs in B cells (8–10). Upon transmission *via* saliva, EBV crosses the mucosal epithelium to infect B cells in the underlying secondary lymphoid tissues, including tonsils (1, 11). As its default program, it expresses latent EBV gene products from circularized viral DNA episomes. In naïve B cells, all eight latent EBV proteins (six nuclear antigens or EBNAs, and two latent membrane proteins or LMPs) plus non-translated RNAs, including EBERs and miRNAs, can be detected (12, 13). This so-called latency III program drives B cells into proliferation and can also be found after *in vitro* immortalization of human B cells by EBV infection, as well as in lymphomas in immune compromised individuals (14). In germinal center B cells, only EBNA1 and the two LMPs are expressed at the protein level and thought to rescue infected cells from deletion without T cell help and B cell receptor engagement during this B cell differentiation stage (15). This latency IIa program is found in Hodgkin’s lymphoma (8). *Via* germinal center differentiation or directly from an early expressed latency IIb program (EBNA without LMP expression), EBV-infected B cells reach the memory B cell pool for persistence (16, 17). In these, EBV shuts down latent protein expression or transiently induces EBNA1 in homeostatically proliferating memory B cells during latency 0 or I, respectively (18). Latency I can also be found in Burkitt’s lymphoma (19). From latency 0 or I, EBV can reactivate upon plasma cell differentiation of its infected B cells into lytic replication and infectious viral particle production (20). Mucosal epithelial cell infection from the basolateral site might then allow for another round of lytic EBV replication prior to shedding into saliva (21). Such epithelial cell infection is thought to be the source for EBV-associated nasopharyngeal and gastric carcinomas, but it remains unclear under which circumstances EBV switches into the associated latency II and I programs in these cells (8). Nevertheless, all tumor-associated EBV infection programs are present in healthy EBV carriers and are thought to be kept in check by immune control.

This immune response is thought to be mainly dependent on cytotoxic lymphocytes, such as CD8^+^ T cells and NK cells, but not antibody responses or antiviral cytokine secretion (22–25). The strongest evidence for this notion comes from primary immunodeficiencies that predispose individuals to EBV-associated pathologies due to single-gene mutations. These identify the perforin/granzyme cytotoxic machinery, T cell receptor signaling, cytotoxic lymphocyte co-stimulation, development of cytotoxic lymphocytes, and their expansion as crucial elements of EBV-specific immune control (22–25). For example, patients with mutations in the co-stimulatory molecule CD27 or its ligand CD70 nearly uniformly suffer from EBV-associated pathologies (26). This co-stimulatory interaction is required for the expansion and cytotoxicity of a subset of EBV-specific CD8^+^ T cells recognizing lytic infection and EBV-specific immune control in mice with reconstituted human immune systems (humanized mice) (27). In the same preclinical model system of EBV infection, depletion of CD8^+^ T cells and NK cells or pharmacological inhibition of predominantly CD4^+^ T cell responses leads to higher viral loads and associated lymphomagenesis carrying primarily the latency III infection program (28–34). These findings suggest that primarily cytotoxic lymphocytes prevent transition of premalignant EBV infection to lymphomagenesis in the vast majority of asymptomatic EBV carriers.

## Burkitt’s Lymphoma and *Plasmodium falciparum*

**Figure 1:**
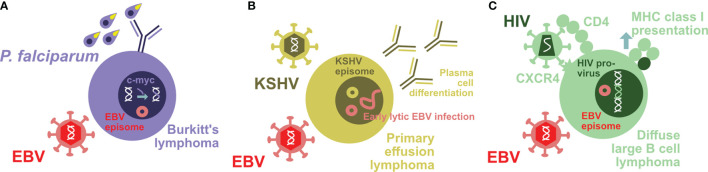
Coinfections modify EBV-associated lymphomagenesis. **(A)** Holoendemic *Plasmodium falciparum* (*P. falciparum*) exposure is associated with endemic Burkitt’s lymphoma. The characteristic c-myc translocation in these uniformly EBV-infected tumor cells might be driven by parasite-induced B cell differentiation. **(B)** Primary effusion lymphoma is to 100% KSHV and to 90% EBV infected. KSHV infection drives the characteristic plasmablastic differentiation of these tumor cells that in turn leads to increased early lytic EBV reactivation. **(C)** HIV is able to infect EBV-transformed B cells by virtue of their CD4 upregulation and maintained CXCR4 expression. The resulting double-infected B cells with integrated HIV provirus upregulate MHC class I antigen presentation and are therefore efficiently eliminated by CD8^+^ T cells.

This balance between lymphomagenesis and its control by the immune system can be disrupted by coinfections. The most prominent case in point is the coinfection with the malaria parasite *Plasmodium falciparum* which primarily infects hepato- and erythrocytes (19, 35, 36). In Sub-Saharan Africa and Papua New Guinea, holoendemic exposure to this parasite is associated with Burkitt’s lymphoma (36, 37). In this endemic form, Burkitt’s lymphoma incidence is highest in the second half of the first decade of life and to 90% associated with EBV. In addition, it carries a characteristic somatic mutation in the form of c-myc translocation into the heavy- or light-chain immunoglobulin locus of the affected B cells (**Figure 1A**). This translocation is thought to occur downstream of the germinal center reaction and its expression of activation-induced deaminase (AID) (38). Germinal center induction and the associated AID expression are promoted by persistent *Plasmodium* infection (39, 40). However, EBV can also directly induce AID expression *via* its latent gene product EBNA3C (41). Thus, Burkitt’s lymphoma, the tumor entity in which EBV was originally discovered (42, 43), carries in its endemic form EBV latency I infection and a c-myc translocation into the immunoglobulin locus. *P. falciparum*, but not other malaria parasites, seems to promote Burkitt’s lymphoma development by driving more infected B cells into a differentiation stage in which c-myc translocation can occur (**Figure 1A**) and by weakening the immune control of the respective tumor cells.

**Figure 2:**
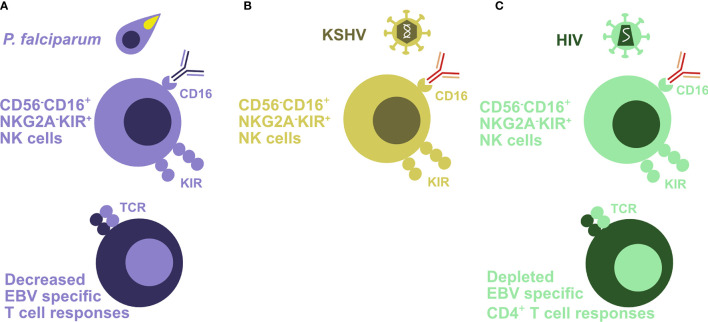
Coinfections attenuate EBV-specific immune control. **(A)** Holoendemic *Plasmodium falciparum* (*P. falciparum*) exposure and especially endemic Burkitt’s lymphoma are associated with an accumulation of terminally differentiated CD56^-^CD16^+^NKG2A^-^KIR^+^ NK cells. In addition, EBV-specific T cell responses are decreased in the affected patients. **(B)** KSHV coinfection drives CD56^-^CD16^+^NKG2A^-^KIR^+^ NK cell differentiation in the presence of EBV infection. **(C)** HIV-infected patients also demonstrate an accumulation of poorly functional CD56^-^CD16^+^NKG2A^-^KIR^+^ NK cells. In addition, this infection depletes EBV-specific CD4^+^ T cells.

Indeed, evidence for weakened immune control of EBV could be found in children with holoendemic *P. falciparum* exposure (44–49). This affects CD4^+^ T, CD8^+^ T, and NK cells. Loss of T cell responses against EBNA1, the sole viral protein that is expressed in latency I and Burkitt’s lymphoma cells, has been documented (47) (**Figure 2A**). But also NK cell differentiation to poorly functional CD56^-^CD16^+^ NK cells has been described in children with Burkitt’s lymphoma (48, 49) (**Figure 2A**) which is thought to compromise NK cell-mediated control of lytic EBV replication (33, 50, 51). NK cell differentiation to CD56^-^CD16^+^ cells could in part be driven by activating killer immunoglobulin-like receptors (KIRs) (52). Higher EBV reactivation and/or diminished immune control leads also to more than 100-fold elevated viral loads in children with holoendemic exposure to *P. falciparum* (53). The development and maintenance of optimal EBV-specific immune control could be compromised by the Th2 environment that is required for immune control of the *P. falciparum* blood stage (54). Indeed, Burkitt’s lymphoma patients carry malaria asymptomatically in Sub-Saharan Africa (55). Why, however, altered EBV-specific immune control and/or increased EBV-infected B cell activation that result in the elevated viral loads of children in holoendemic malaria regions are particularly associated with the *P. falciparum* parasite remains unclear. Unfortunately, an *in vivo* model to probe the interaction of these two important human pathogens is still lacking.

## Primary Effusion Lymphoma and KSHV

Another coinfection that modifies both EBV-associated tumor cells and EBV-specific immune control is the Kaposi sarcoma-associated herpesvirus (KSHV) (56). This human γ-herpesvirus that is closely related to EBV is associated with the tumors Kaposi sarcoma and primary effusion lymphoma (PEL) (57). PEL cells are 100% KSHV infected and carry simultaneously in 90% of cases EBV in the very same lymphoma cells (9, 58). In most cases, EBV infection displays latency I in these tumors. PEL are also the only tumor entity in which the KSHV genome is maintained after culturing *in vitro* (59). EBV enhances KSHV genome maintenance in these PEL cell lines (60). Accordingly, EBV supports KSHV persistence after human B cell infection *in vivo* in humanized mice (61, 62) and *in vitro* (63, 64) but needs to occur at nearly the same time to maintain KSHV. Probably as a result, KSHV infection is not found without EBV coinfection in African populations from Cameroon or Uganda (65, 66). In these studies, EBV infection was even identified as the most significant environmental factor for KSHV infection. Similarly, the orthologue viruses can be transmitted together in monkeys (67). Thus, EBV supports KSHV persistence.

Vice versa KSHV, however, also modifies EBV-associated lymphomagenesis and its immune control. PELs have a characteristic plasmablastic phenotype with a distinct gene expression pattern compared to solely EBV latency III carrying lymphomas (68). Accordingly, not only does KSHV coinfection increase lymphomagenesis in humanized mice, but also the resulting double-infected B and plasma cells show the characteristic gene expression pattern of PELs (61, 62) (**Figure 1B**). According to this plasmablastic differentiation, *in vivo* coinfected B cells also reactivate EBV at higher frequency into lytic infection (**Figure 1B**), and this can also be demonstrated in patient-derived PEL sections (61). This higher lytic EBV replication contributes to the increased lymphomagenesis of KSHV- and EBV-coinfected humanized mice, because coinfection with a lytic replication-deficient ΔBZLF1 EBV virus abolishes the increased tumor formation (61, 62). Indeed, the influence of lytic EBV replication on virus-associated tumorigenicity has also been observed in other settings with decreased lymphoma formation by ΔBZLF1 EBV infection alone or increased tumor frequency due to BZLF1 overexpression (69–72). In addition to regulating EBV-associated lymphomagenesis, KSHV coinfection also influences immune compartments (62). In both coinfected humanized mice and Kenyan children, CD56^-^CD16^+^CD38^+^CXCR6^+^ NK cells are enriched (**Figure 2B**). This terminal NK cell differentiation stage retains only limited abilities to expand, secrete cytokines, or kill infected cells (62), while early differentiated CD56^+^CD16^+^NKG2A^+^KIR^-^ NK cells are the protective entity against lytic EBV replication and expand during infectious mononucleosis (33, 51, 73, 74). These studies suggest that KSHV influences EBV-associated lymphomagenesis by plasmablastic differentiation of coinfected B cells, associated induction of early lytic EBV replication, and compromising immune control of lytic EBV infection by causing NK cell differentiation to the less functional and weakly protective terminally differentiated CD56-negative subpopulation.

## B Cell Lymphomas and HIV

PEL like most other EBV-associated lymphomas increase also during coinfection with the human immunodeficiency virus (HIV) (9, 14, 75). Primarily, loss of EBV-specific immune control upon HIV-mediated CD4^+^ T cell depletion and associated CD8^+^ T cell senescence are thought to contribute to this increase in EBV-associated malignancies. Accordingly, the frequency of EBV association is increased in Burkitt’s lymphoma, Hodgkin’s lymphoma, diffuse large B cell lymphoma, PEL, and primary CNS lymphoma in HIV-infected patients compared to patients without coinfections by HIV, KSHV, or *P. falciparum* (75). Accordingly, the selective loss of T cell responses to EBV antigens was found during progression to EBV-associated lymphomas in HIV-infected patients (76–78) (**Figure 2C**). The earlier occurrence of Burkitt’s and Hodgkin’s lymphoma and later emergence of EBV latency III lymphomas might indicate that progressive loss of T cell-mediated immune control against EBV allows more and more immunogenic virus-induced lymphomas to emerge. Only the frequencies of the latter have been significantly lowered by anti-retroviral therapies (ART) (14). Along these lines, T cell responses against the EBV latency I antigen, particularly in healthy virus carriers frequent EBNA1-specific CD4^+^ T cells (79, 80), seem to be lost prior to EBV-associated lymphoma development in HIV patients (76). Similarly, the protective function of CD8^+^ T cell-mediated immune control of EBV is lost during coinfection with HIV in humanized mice (31). While antibody-mediated CD8^+^ T cell depletion increased EBV viral loads in single infected humanized mice, the already increased EBV titers during HIV coinfection could not be further increased by CD8^+^ T cell depletion (31). Furthermore, HIV has also been described to differentiate NK cells to the less protective CD56^-^CD16^+^ phenotype (81–85) (**Figure 2C**). As discussed above, this terminal NK cell differentiation stage accumulates at the expense of early differentiated CD56^+^CD16^+^NKG2A^+^KIR^-^ NK cells that target lytic EBV replication (51). Thus, HIV coinfection compromises EBV-specific immune control by T and NK cells, resulting in increased frequencies of EBV-associated lymphomas of all EBV latency patterns.

However, frequencies of some, mostly low immunogenic EBV-associated lymphomas remain high after CD4^+^ T cell count stabilization due to anti-retroviral treatment (ART) in HIV patients (14). Therefore, chronic inflammation due to HIV infection during ART or a more direct effect of HIV might continue to promote Burkitt’s and Hodgkin’s lymphoma despite restored general immune control of EBV against latency III-associated lymphomas. Along these lines, EBV-transformed B cells can be directly infected by HIV (31). During B cell infection, EBV upregulates CD4 on already CXCR4-expressing cells, and X4 tropic HIV strains can establish infection as well as reversely transcribed viral DNA integration in EBV-transformed B cells (31) (**Figure 1C**). Even under conditions of reduced CD8^+^ T cell function in HIV plus EBV-coinfected humanized mice, these double-infected B cells are efficiently cleared, presumably in part due to the upregulation of the antigen-processing machinery for MHC class I presentation in these cells (31) (**Figure 1C**). However, it is tempting to speculate that the HIV-induced expression of DNA-modifying enzymes, such as the APOBEC family of cytidine deaminases (86), might promote somatic mutations that are required for lymphomas with EBV latency I and II expression patterns to emerge. Thus, also HIV might contribute to EBV-associated lymphomagenesis by both immune suppression and modifying EBV-induced tumor cells directly.

## Other Infections and EBV

Despite the near perfect immune control of EBV in the vast majority of asymptomatic virus carriers, it is associated with around 1% of all human tumors (8, 87). Most of these are, however, nasopharyngeal (NPC) or gastric carcinomas (GC), and not lymphomas (87). Unfortunately, it is quite unclear under which circumstances EBV establishes growth-transforming latent infection in epithelial cells, because this cannot yet be modeled *in vitro* and the only non-malignant EBV infection in the oropharyngeal epithelium, termed hairy leukoplakia, is caused by lytic EBV infection (88). However, it is assumed that premalignant genetic lesions that are introduced into the respective epithelial cells by carcinogenic substances in the diet and/or possible chronic inflammation in the oropharyngeal cavity or stomach provide fertile grounds for epithelial cell transformation by EBV, resulting in latency II NPC or latency I GC (89). One such coinfection that promotes premalignant genetic lesion development in the gastric epithelium could be *Helicobacter pylori* infection whose coinfection with EBV has been reported in GC patients (90, 91). The induced mutations are thought to allow EBV to establish latent transforming infection in epithelial cells. In addition to generating chronic inflammation, *H. pylori* might also directly promote EBV-driven proliferation in gastric epithelial cells by downregulating tumor suppressors (92). In contrast, coinfection of EBV with human papillomavirus (HPV) that is also associated with NPC is rare (93). Thus, in addition to *P. falciparum*, HIV, and KSHV, other coinfections might induce genetic lesions *via* chronic inflammation, particularly in epithelial cells, that are then explored by EBV during virus-associated carcinogenesis.

## Conclusions and Outlook

More than 55 years after the discovery of EBV in Burkitt’s lymphoma (42, 43), it is clear that coinfections are some of the strongest environmental modulators of EBV-associated pathology. However, the mechanisms underlying these association remain largely unclear and require both clinical studies on affected patients as well as preclinical model systems to study coinfections. Along these lines, humanized mice offer the possibility to study lymphotropic coinfections such as KSHV or HIV together with EBV (31, 61, 62). In these instances, coinfections were able to regulate both lymphomagenesis in the EBV-infected B cells as well as their immune control. However, coinfections outside the hematopoietic lineage, such as for example in human hepatocytes, remain challenging (94–98). This includes *P. falciparum* for which both human hepatocytes and erythrocytes are required to complete its life cycle. Thus, endemic Burkitt’s lymphoma remains an enigma more than 60 years after its first description (19, 99–101). Nevertheless, the studies summarized in this review document that infections such as by the human tumor virus EBV never occur in isolation in humans. This suggests that also our preclinical animal models need to be adapted with coinfections in order to more faithfully recapitulate human physiology. Some previously established paradigms of microbiology and immunology might need to be revisited in such coinfection models.

## Author Contributions

The author confirms being the sole contributor of this work and has approved it for publication.

## Funding

Research in the author’s laboratory is funded by Cancer Research Switzerland (KFS-4962-02-2020), KFSP-Precision MS and HMZ ImmunoTargET of the University of Zurich, the Cancer Research Center Zurich, the Vontobel Foundation, the Sobek Foundation, the Swiss Vaccine Research Institute, Roche, Novartis, Innosuisse, and the Swiss National Science Foundation (310030_204470/1, 310030L_197952/1, and CRSII5_180323). The funders were not involved in the study design, collection, analysis, interpretation of data, the writing of this article, or the decision to submit it for publication.

## Conflict of Interest

The author declares that the research was conducted in the absence of any commercial or financial relationships that could be construed as a potential conflict of interest.

## Publisher’s Note

All claims expressed in this article are solely those of the authors and do not necessarily represent those of their affiliated organizations, or those of the publisher, the editors and the reviewers. Any product that may be evaluated in this article, or claim that may be made by its manufacturer, is not guaranteed or endorsed by the publisher.
